# High-density lipoproteins attenuate high glucose-impaired endothelial cell signaling and functions: potential implications for improved vascular repair in diabetes

**DOI:** 10.1186/s12933-017-0605-8

**Published:** 2017-09-29

**Authors:** Xing Chen, My-Ngan Duong, Peter J. Psaltis, Christina A. Bursill, Stephen J. Nicholls

**Affiliations:** 1 Department of Cell Biology and Cardiovascular Medicine, Cleveland Clinic, Cleveland, OH 44195 USA; 2 Heart Health, South Australian Health and Medical Research Institute, Adelaide, SA 5000 Australia; 3 South Australian Health and Medical Research Institute, PO Box 11060, Adelaide, SA 5001 Australia

**Keywords:** HDL, Hyperglycaemia, Endothelial cells, Atherosclerosis

## Abstract

**Background:**

Abnormalities of endothelial cell function are proposed to be a critical factor underlying adverse cardiovascular outcomes in the setting of hyperglycaemia. While high-density lipoproteins (HDL) have been demonstrated to be cardioprotective, the impact on the endothelium in hyperglycaemia has not been fully elucidated.

**Methods:**

Human umbilical vein endothelial cells (HUVECs) were exposed to high-glucose conditions using dextrose, the main isoform of glucose, and native HDL. HUVEC proliferation and migration were determined. The key signalling pathways that regulate endothelial cell function were also characterized.

**Results:**

Increasing concentrations of dextrose resulted in significant reductions in HUVEC proliferation, this was attenuated by coincubation with HDL. In support of this, HDL was also found to rescue dextrose impaired expression of PCNA and the activation (phosphorylation) of the key transcription factor for proliferation ERK. Dextrose also dose-dependently inhibited HUVEC migration, which was mitigated by co-incubation with HDL. Consistent with this, HDL prevented dextrose-induced inhibition of p38 phosphorylation, responsible for cell migration. Finally, phosphorylation of the pro-survival transcription factor Akt was dose-dependently inhibited by dextrose, however, this was completely rescued by co-administration with HDL.

**Conclusion:**

Dextrose-induced hyperglycaemia causes the impairment of endothelial cell proliferation and migration and inhibits the activation of ERK, p38 and Akt pathways. The protective effects of HDL in this milieu highlights the potential for HDL to improve vascular repair in patients with impaired glucose homeostasis.

## Introduction

Uncontrolled hyperglycaemia has long been recognized as a major risk factor in the development of vascular complications in diabetic patients [1]. Diabetic macrovascular complications are involved in ischemic heart disease [2], peripheral vascular disease [3] and thromboembolic stroke [4]. These complications are major contributors to the morbidity and mortality associated with diabetes [5]. Another important diabetic feature are vascular lesions which involves impaired endothelial-dependent vasomotor responses and significant alterations in endothelial growth, survival and migration induced by exposure of vascular endothelial cells to high glucose [6–8]. A causal link between diabetic hyperglycaemia and the development of macrovascular complications has been established early in the disease onset and tight glucose control in diabetic patients have been shown to reduce the progression of disease [9].

Endothelial cells play critical roles in vascular biology, being both the protective inner lining of vessels and the local site for oxygen delivery to all tissues. Endothelial damage or dysfunction is considered a critical initiator of large vessel diseases such as atherosclerosis [8]. An intact endothelial cell monolayer modulates local haemostasis and thrombolysis and provides a non-permeable barrier protecting vascular smooth muscle cells (VSMCs) from circulating growth-promoting factors [10]. Vascular endothelial cell proliferation and migration are vital in many physiological and pathological processes, such as angiogenesis and healing of the injured endothelium [11].

It has been shown in previous investigations that high glucose exposure in human umbilical vein cells (HUVEC) impairs endothelial function such as insulin signalling [12] and expression of a host of proteins involved in thrombosis and blood viscosity [13]. A number of in vitro studies focused on the effects of high glucose concentration on growth and survival of various types of endothelial cells (EC), including HUVECs [7, 14], human pulmonary artery EC [15], human dermal microvascular EC [16], aortic EC [17] and retinal EC [18, 19]. However, conflicting results in EC properties under high glucose [20, 21] make the interpretation of these results difficult. These conflicting reports may be explained by differences in species, macrovascular ECs versus microvascular ECs, or changes in experimental conditions, but most likely reflects fundamental differences in EC cell type.

Epidemiological studies show that high-density lipoprotein (HDL) is antiatherogenic and an independent protective factor for coronary artery diseases [22]. HDL and its major protein constituent apolipoprotein A-1 (apoA-1) play major roles in mediating reverse cholesterol transport (RCT), an important atheroprotective mechanism. HDL has many other functions, including removal or detoxification of oxidized sterols/phospholipids and its anti-inflammatory, antioxidant and antithrombotic activities [23–25]. HDL also exhibits potent endothelial protective and reparative capabilities [10, 24]. HDL has been shown to promote endothelial cell migration and protect it from cellular apoptosis as well as elevate nitric oxide (NO) production through increases in endothelial NO synthase (eNOS) expression and activity [10, 26]. However, the efficiency of HDL on high glucose-triggered dysfunction in ECs and the related signalling pathways is still to be fully elucidated.

The present study aimed to determine if HDL can attenuate dextrose-induced high glucose impaired HUVEC proliferation and migration as well as the activation of the key transcription factors ERK, p38 and Akt that regulate these functions. We used dextrose as this is the most common, naturally occurring isoform of glucose. We report that dextrose inhibits EC migration, proliferation and the phosphorylation of ERK, p38 and Akt. Coincubation with HDL completely mitigates these effects. Our findings provide a greater understanding of the endothelial protective effects of HDL, with implications for the treatment of diabetic vascular complications.

## Materials and methods

### Cell isolation, culture and preparation of HDL

HUVECs (Cell Applications Inc., San Diego, CA, USA) were maintained in MCDB (Molecular, Cellular, and Developmental Biology) medium containing 15% fetal bovine serum (FBS, lipoprotein deficient), 0.009% heparin, 0.015% endothelial cell growth supplement (Cat. Number E0760; Sigma, St. Louis, MO, USA) and used between passage 3 and 6 [10]. HDL (1.063 < d < 1.21 g/mL) was isolated by sequential ultracentrifugation from human plasma as previously described [27]. Briefly, the density of human plasma was modified with potassium bromide (Sigma, St. Louis, MO, USA) to the desired density and sequentially ultracentrifuged in a Beckmann 50.2 Ti rotor (Beckmann Coulter, Brea, CA, USA) at 50,000 rpm at 4 °C. The HDL fraction was dialysed against 3 × 1 L PBS, filtered-sterilised (0.22 µm) and stored in a light-excluding container at 4 °C under nitrogen gas. HDL protein was measured using a Bio-Rad protein assay (Bio-Rad, Richmond, CA, USA) and added to the HUVECs on the basis on total HDL protein concentration (µg/mL).

### Cell proliferation assay

Proliferation assays were performed in plates coated with collagen II (Cat. Number C9301; Sigma, St. Louis, MO, USA). Briefly, HUVECs were serum-deprived for 24 h (0.5% FBS) before the cells were exposed to increasing dextrose concentrations (5.7–40 mM; Cat. Number G7528; Sigma, St. Louis, MO, USA) and/or HDL (5–120 μg/mL total protein concentration) in a background media with 2.5% FBS. Cell proliferation was measured after a 96 h incubation as cell number using a bromodeoxyuridine (BrdU) incorporation assay (BrdU kit; Millipore, Temecula CA, USA). Total cellular protein content in 6-well plates was determined from cell lysates with the Bio-Rad protein assay (Bio-Rad, Richmond, CA, USA) [7]. The experiments were performed in triplicate and repeated at least 3 times.

### Boyden chamber migration assay

A migration assay was performed using transwells (8 µm pore polycarbonate membrane, 24 well, Costar, Sigma, St. Louis, MO, USA) [10, 28]. Briefly, after HUVECs were seeded on the upper chamber, they were firstly serum-deprived for 12 h (0.5% FBS) then the transwells were placed into the lower chamber containing increasing dextrose concentrations (5.7–40 mM) and/or HDL (80 μg/mL in total protein concentration) in a background media of 2.5% FBS and incubated overnight. The cells that migrated to the underside of the membrane were fixed and the membranes were mounted on slides using mounting medium with DAPI (Vector Laboratories, Burlingame, CA, USA.). The number of migrated cells was determined from microscopic images of transwell membranes. The experiments were performed in triplicate and repeated at least 3 times.

### Western blotting

HUVECs were seeded in 6-well plates for 48 h in MCDB medium containing 15% FBS, after which the cells were serum-starved (0.5% FBS in MCDB) for 24 h. The cells were then incubated with dextrose or HDL (2.5% FBS background media) at the indicated concentrations for either: 72 h (PCNA), 5 min (pAkt/Akt) or 15 min (p-p38/p38 and pERK/ERK). Cells were lysed, sonicated and subjected to standard Western blotting methods [10, 29]. The individual primary antibodies used were anti-PCNA (1:2000 dilution), anti-Akt/anti-pAkt (residue Ser-473, 1:1000), anti-ERK/anti-p-ERK1/2 (1:2000) and anti-p-p38/anti-p38 (1:1000) (Cell Signaling, Beverly, MA). Equal protein loading was verified by stripping membranes of original antibodies and re-probing with the primary antibody anti-β-actin (1:3000; Cell Signaling, Beverly, MA). Protein levels were quantified from digitized images using ImageLab (Bio-Rad, Richmond, CA, USA). The inhibitors PD98059 (ERK1/2 inhibition; 6.0 µM; Sigma, St. Louis, MO, USA), SB203580 (p38 inhibition; 50 µM; Sigma, St. Louis, MO, USA) and LY294002 (Akt inhibition; 20 µM: Sigma, St. Louis, MO, USA) were dissolved in DSMO (dimethyl sulfoxide; Sigma, St. Louis, MO, USA) and were used to identify the pathway of cellular activation.

### Data analysis

Data are expressed as mean ± SEM unless stated otherwise. Means of 2 groups were compared using Student’s *t* test (unpaired, 2-tailed), and one-way ANOVA was used for comparison of more than 2 groups, with *p* < 0.05 considered to be statistically significant. These results were tested for normality and equality of variances. For those results that did not satisfied those criteria, non-parametric Wilcoxon or Welch t test were used. Unless indicated in the figure legends, all the experiments were performed at least 3 times in triplicate.

## Results

### HDL attenuates dextrose-induced inhibition of HUVEC proliferation

**Fig. 1:**
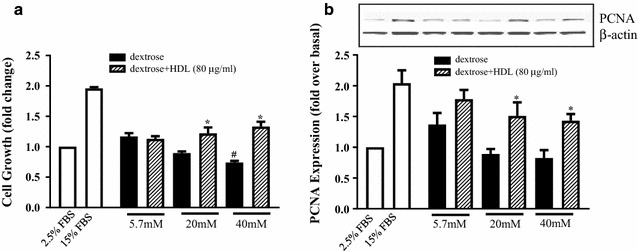
HDL attenuates dextrose-induced inhibition of HUVEC proliferation. **a** Cell proliferation was determined after cells were incubated with dextrose (5.7, 20 and 40 mM) or dextrose plus HDL (80 µg/mL) for 96 h using BrdU incorporation. 2.5% FBS (control) and 15% FBS (positive control) treatments were also included. **b** Protein levels of PCNA were determined in HUVEC lysates incubated with dextrose (5.7, 20 and 40 mM) or dextrose plus HDL (80 µg/mL) for 72 h using Western blotting. β-Actin was used as the protein loading control. Data shown are the mean ± SEM of results from three independent experiments. ^#^p < 0.05 relative to the respective control (cells in MCDB medium containing 2.5% FBS). *p < 0.05 relative to the dextrose only treatments at the same concentration

Treatment of HUVECs with dextrose resulted in a significant concentration-dependent decrease in BrdU proliferation (22.1 ± 5.1% at 20 mM and 36.5 ± 6.1% at 40 mM, Fig. 1a), compared to the normo-glucose control (5.7 mM dextrose). This inhibition of proliferation was reversed when HDL (80 µg/mL) was co-incubated with dextrose, increasing proliferation when compared to dextrose only control cells at each concentration (24.3 ± 7.6 and 33.5 ± 5.9% at 20 and 40 mM dextrose respectively, Fig. 1a). Consistent with the proliferation findings, dextrose-induced high glucose caused dose dependent reductions in PCNA (proliferating cell nuclear antigen) protein levels. Co-incubation with HDL, however, was able to completely mitigate dextrose-induced reductions in PCNA (Fig. 1b).

### HDL attenuates dextrose-induced inhibition of ERK activation

**Fig. 2:**
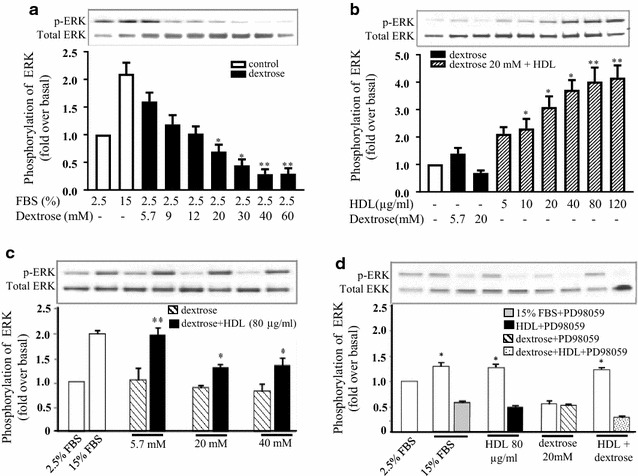
HDL attenuates dextrose-induced inhibition of ERK activation. HUVECs were incubated with increasing dextrose concentrations (5.7–60 mM) for 15 min (**a**), or HDL (5–120 µg/mL) (**b**), *p < 0.05, **p < 0.01 relative to dextrose alone (5.7 mM). Incubation of HDL (80 µg/mL) with increasing dextrose (5.7, 20 and 40 mM) in a background media of 2.5% MCDB, *p < 0.05, **p < 0.01 compared to relative dextrose concentration alone (**c**). HDL (80 µg/mL) with either dextrose (20 mM) and/or ERK inhibitor (PD98059) for 1 h (**d**). ERK phosphorylation was determined by Western blot analysis. Data is expressed as a ratio of p-ERK to total-ERK. *p < 0.05 relative to the PD98059 treatment. Data shown are representative of results from 3 independent experiments

Changes in the activation of ERK1/2, a key signalling protein for the induction of proliferation, following incubation with dextrose and HDL were next investigated. Dextrose (5.7–60 mM) inhibited the phosphorylation of ERK1/2 in a step-wise manner (Fig. 2a). Suppression of ERK1/2 activation by dextrose (20 mM) was completely reversed with the co-incubation of HDL (5–120 µg/mL). ERK1/2 phosphorylation was significantly elevated above normo-glucose control levels at HDL concentration of 10 µg/mL, with maximal ERK1/2 phosphorylation (~ fourfold) observed at 120 µg/mL HDL (Fig. 2b). It was found that HDL (80 µg/mL) was able to overcome dextrose-induced inhibition of ERK1/2 activation with higher doses of dextrose (up to 40 mM, Fig. 2c). To confirm the specific induction of ERK1/2 activation by HDL in the presence of dextrose, the effects of ERK1/2 inhibitor, PD98059, were assessed. Co-incubation with PD98059 completely abrogated FBS, HDL and dextrose + HDL—induced ERK1/2 phosphorylation, confirming the involvement of HDL in this pathway (Fig. 2d).

### HDL rescues dextrose-induced inhibition of HUVEC migration

**Fig. 3:**
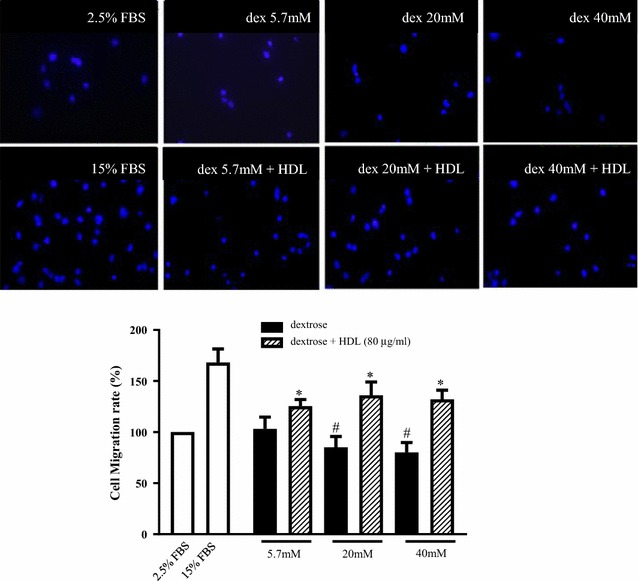
HDL rescues dextrose-induced inhibition of HUVEC migration. HUVEC migration was determined using transwell membranes. HUVECs were seeded on the upper chamber, serum-deprived for 12 h, then the transwells were placed into the lower chamber containing increasing dextrose concentrations (5.7–40 mM) and/or HDL (80 μg/mL) and incubated overnight. The cells that migrated through the membrane were fixed and stained by DAPI. Upper panels are representative images of membranes of migrated cells. Data shown are the mean ± SEM of results performed in triplicate from 3 independent experiments. ^#^p < 0.05 relative to the control (2.5% FBS treatment). *p < 0.05 compared to relative dextrose concentration alone

Dextrose-induced high glucose inhibited HUVEC migration in a concentration dependent manner, with a decrease in HUVEC migration of 23.6 ± 5.7 and 38.7 ± 7.1% at dextrose concentrations of 20 and 40 mM respectively. Co-incubation with HDL (80 µg/mL) was able to reverse this inhibition and caused increases in HUVEC migration, compared to dextrose only controls at the respective concentrations (21.7 ± 2.3, 55.5 ± 6.5 and 60.8 ± 3.3% at 5.7, 20 and 40 mM dextrose respectively, Fig. 3).

### HDL attenuates dextrose-induced inhibition of p38 activation

**Fig. 4:**
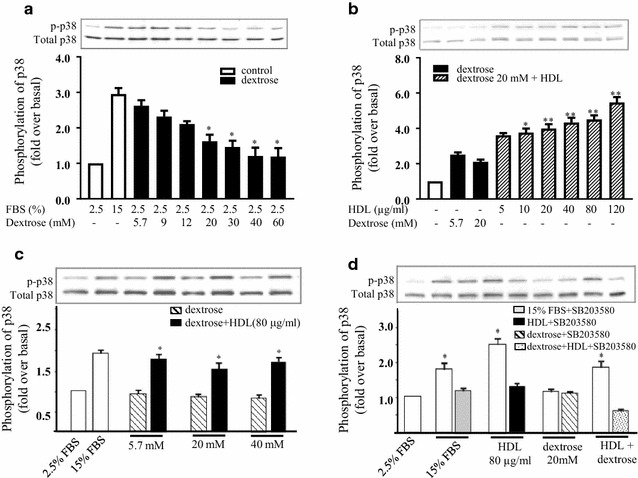
HDL attenuates dextrose-induced inhibition of p38 activation. HUVECs were incubated with increasing dextrose concentrations (5.7–60 mM) for 15 min (**a**) or HDL (5–120 µg/mL) (**b**), *p < 0.05, relative to dextrose alone (5.7 mM). Incubation of HDL (80 µg/mL) with increasing dextrose (5.7, 20 and 40 mM), *p < 0.05, **p < 0.01 compared to relative dextrose concentration alone (**c**). HDL (80 µg/mL) with either dextrose (20 mM) and/or p38 inhibitor (SB203580) for 1 h (**d**). p38 phosphorylation was determined by Western blot analysis. Data is expressed as a ratio of p-p38 to total-p38. *p < 0.05 relative to the SB203580 treatment. Background media was 2.5% FBS MCDB. Data shown are representative of results from 3 independent experiments

Changes in the activation of p38, a critical promoter of cell migration, was next assessed. Dextrose (5.7–60 mM) inhibited the phosphorylation of p38 in a dose-dependent manner (Fig. 4a). In the presence of 20 mM dextrose, co-incubation with HDL at a concentration as low as 10 µg/mL was able to activation p38 phosphorylation above the normo-glucose control, with maximal p38 phosphorylation (~ 5.5-fold) observed at 120 µg/mL HDL (Fig. 4b). HDL (80 µg/mL) was also able to increase p38 phosphorylation in higher concentrations of dextrose (up to 40 mM, Fig. 4c). Finally, to confirm the activation of p38 by HDL, a specific inhibitor of p38, SB203580, was included. It was found that incubation with SB203580 attenuated FBS, HDL and dextrose + HDL-induced p38 phosphorylation, confirming the role of HDL in the activation of this pathway.

### HDL mitigates the inhibition of Akt phosphorylation by dextrose

**Fig. 5:**
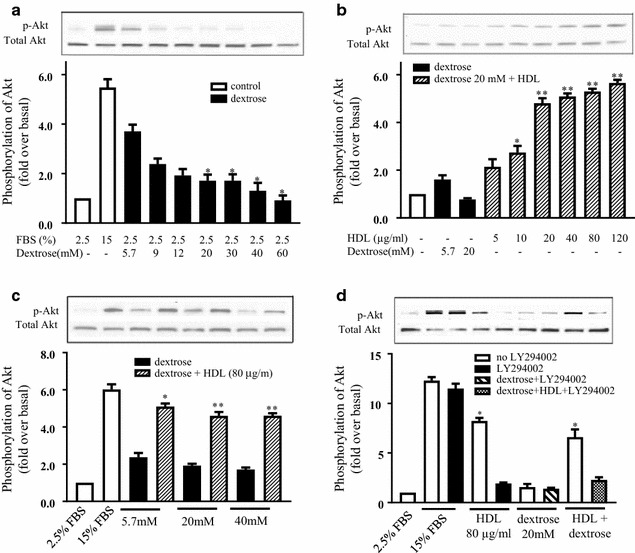
HDL mitigates the inhibition of Akt phosphorylation by dextrose. HUVECs were incubated with increasing dextrose concentrations (5.7–60 mM) for 15 min (**a**) or HDL (5–120 µg/mL) (**b**), *p < 0.05, **p < 0.01 relative to dextrose alone (5.7 mM). Incubation of HDL (80 µg/mL) with increasing dextrose (5.7, 20 and 40 mM), *p < 0.05, **p < 0.01 compared to relative dextrose concentration alone (**c**). HDL (80 µg/mL) with either dextrose (20 mM) and/or pAkt inhibitor (LY294002) for 1 h (**d**). Akt phosphorylation was determined by Western blot analysis. Data is expressed as a ratio of p-Akt to total-Akt. *p < 0.05 relative to the LY294002 treatment. Data shown are representative of results from 3 independent experiments

Increasing concentrations of dextrose caused a step-wise reduction in Akt phosphorylation (Fig. 5a), when compared to the normo-glucose control. In the presence of dextrose (20 mM), HDL was able to significantly increase the phosphorylation of Akt that reached significance at the 10 µg/mL concentration and increased further at 20 µg/mL, followed by a plateau, but remained significantly elevated up to 120 µg/mL, compared to dextrose (20 mM) only control cells (Fig. 5b). Furthermore, co-incubation of dextrose with HDL (80 µg/mL) was able to rescue high glucose-impaired Akt phosphorylation at dextrose concentrations up to 40 mM (Fig. 5c). A specific inhibitor of Akt, LY294002, was used to confirm that activation of this pathway by HDL. Inclusion of LY294002 was found to completely prevent HDL-induced activation of Akt phosphorylation (Fig. 5d).

## Discussion

We report that HDL is able to attenuate high glucose-impaired endothelial cell function and signalling. Our studies show that dextrose-induced high glucose significantly suppresses the important endothelial functions of proliferation and migration, as well as the related ERK, p38 and Akt signalling pathways. Coincubation with HDL is able rescue these impairments to normo-glucose levels. These findings have implications for the therapeutic modulation of endothelial repair by HDL in diabetes.

The cardio-protective role of HDL has been observed for decades [22–25], but there are gaps in knowledge regarding its effect on HUVEC proliferative and the related cell signalling pathway in the settling of early high glucose insult. The protein kinase Akt is a multifunctional regulator of cell growth and survival [30, 31]. Akt is primarily activated when the threonine 308 (Thr^308^) and serine 473 (Ser^473^) residues are phosphorylated by PI3-K, which in turn then activate Akt serine/threonine kinase activity. In this study, the level of phosphorylated Akt at Ser^473^ was significantly inhibited by dextrose-induced high glucose, but rescued by coincubation with HDL. Pre-treatment of HUVECs with the Akt inhibitor LY294002 almost completely inhibited HDL-induced Akt phosphorylation providing further evidence that this is mediated by Akt. These findings are consistent with other studies that have found reconstituted HDL (rHDL) augments Akt phosphorylation [26]. In the early stages of a high glucose insult, suppression of the activation of Akt likely plays an important role in the impairment of cell survival [32]. It has been shown that hyperglycaemic exposure results in decreased viability and attenuated proliferation of endothelial cells and this is the result of downregulation of platelet-derived growth factor C and its receptor [33]. The activation of Akt is downstream of this axis. Therefore, the maintenance of Akt by HDL in high glucose suggests HDL may play a role in improved endothelial integrity in hyperglycaemia. This concept is consistent with previous studies [6, 34].

The processes of endothelial cell proliferation and migration are crucial to both neovascularisation and a successful response to vascular injury [9]. High glucose induced endothelial dysfunction is known to not only involve impaired endothelial cell proliferation but also cell migration. ERK and Akt (i.e. MAPK) pathways promote endothelial cell proliferation and migration in response to various extracellular stimuli [35]. Major subfamilies of structurally related MAPKs have been identified in mammalian cells, including ERK1/2 MAPK, p38 MAPK and c-Jun N-terminal kinase/stress-activated protein kinase (JNK/SAPKs) [11]. It has been shown that HDL-stimulated endothelial cell migration is driven by the activation of Src kinase, PI3K-kinase and p44/42 MAP kinase [26]. The current study now shows that HDL rescues the high-glucose-impaired HUVECs migration. Consistent with this we found that HDL reversed the inhibition of p-38 activation in high glucose, a key transcription factor for the promotion of cell migration. The use of a specific p38 inhibitor in this study attenuated the induction of p-p38 by HDL in high glucose, confirming the importance of its role. These results demonstrate a role for HDL in the rescue of high glucose-impaired cell migration and proliferation. In support of our findings, a recent study [36] found rHDL was able to restore angiogenesis in a diabetic murine model of hind limb ischemia. This was shown in vitro to be mediated by the scavenger receptor (SR-BI) which led to the activation of the PI3 K/Akt signalling pathway. On the flip side, dysfunctional HDL isolated from diabetic patients, has diminished capacity to stimulate HUVECs proliferation and migration [37]. These investigators showed that dysfunctional HDL induced Akt phosphorylation initially but this was attenuated with time as SR-BI was down regulated by the dysfunctional lipoprotein. Glycated HDL was also shown to attenuate NO production and increase reactive oxygen/nitrogen species in human aortic endothelial cells [38]. Other properties of HDL were also compromised when HDL is glycated, including its antioxidant and anti-inflammatory properties [39].

Clinical studies using infusions of reconstituted HDL (rHDL, apoA-1 + phospholipid) have already demonstrated promising findings in diabetic patients. For example, a single infusion of rHDL into type 2 diabetes mellitus (T2DM) patients was found to reduce platelet activation [40]. Other studies have found that rHDL infusions in T2DM patients elevate circulating endothelial progenitor cell number [41] and increase the anti-inflammatory properties of endogenous HDL [42]. These studies report that the kinetics of an rHDL infusion are such that there is a steady increase in circulating HDL-cholesterol (50%) and apoA-1 concentrations (> twofold) during the 4 h infusion period. Then out to 72 h post-infusion, the concentration of apoA-1 drops by ~ 50% while the HDL-cholesterol remains elevated. These studies suggest that in the context of diabetes, infusions of rHDL are likely to provide beneficial effects on the endothelium. Whilst the half-life of apoA-1 and HDL-cholesterol may be viewed as relatively short, it must sufficient to impart significant changes during that time.

This interaction between HDL, SR-BI, endothelial function and its signalling pathways may have important implications not only in CVD but also in cancer [43]. Depending on the cancer type, SR-BI expression can correlate with survival rates. SR-BI activation by HDL play a critical role in signalling that stimulates endothelial cell proliferation and migration, important for tumour growth. HDL have been shown to activate Akt and ERK1/2 pathways in breast cancer while knockdown and pharmacological inhibition of SR-BI resulted in a decrease in these pathways [44].

In conclusion, we show that HDL protects endothelial cells from high glucose-impaired cell proliferation and migration. Additionally, HDL rescues high glucose-impaired activation of ERK, p38 and Akt signalling pathways. These findings with HDL suggest that it could be considered as a future therapeutic target to protect against diabetic vascular complications.

## Abbreviations

EC: endothelial cells
HDL: high density lipoproteins
VSMC: vascular smooth muscle cells
HUVECs: human umbilical vein endothelial cells
ApoA-1: apolipoprotein A-1
RCT: reverse cholesterol transport
NO: nitric oxide
eNOS: endothelial nitric oxide synthase
PCNA: proliferating cell nuclear antigen
MAPK: mitogen-activated protein kinases
ERK: extracellular signal-regulated kinases
PI3-K: phosphatidylinositide 3-kinases
MCDB: molecular, cellular, and developmental biology media
FBS: foetal bovine serum
BrdU: bromodeoxyuridine
HG: high glucose
